# Loneliness and Alzheimer’s disease: Testing associations using univariable and multivariable Mendelian randomisation models

**DOI:** 10.1002/alz.091264

**Published:** 2025-01-09

**Authors:** Roopal Desai, Verena Zuber, Amber John, Natalie L Marchant, Georgina Charlesworth, Joshua Stott

**Affiliations:** ^1^ University College London, London United Kingdom; ^2^ Imperial College London, London United Kingdom; ^3^ University College London, London, UK United Kingdom; ^4^ Division of Psychiatry, University College London, London United Kingdom; ^5^ North East London Foundation Trust, London United Kingdom; ^6^ Department of Clinical, Educational, and Health Psychology, Division of Psychology and Language Sciences, University College London, London United Kingdom

## Abstract

**Background:**

Social isolation, encompassing factors like living alone and limited social contact, is associated with increased risk of Alzheimer’s disease (AD). One theory is that loneliness, which is the negative psychological affect often associated with social isolation, mediates the relationship between social isolation and AD. Mendelian randomization (MR) approaches that have thus far been used to investigate causal relationships between loneliness and dementia risk have not supported results from observation studies. However, loneliness has shared genetic overlap with other traits which are independently associated with dementia. The presence of these pleiotropic risk factors may obscure the results of univariable MR analysis. Therefore, the aim of the current study was to better understand possible causal pathways between loneliness and dementia by including multiple pleiotropic risk factors, specifically educational attainment, alcohol dependence, neuroticism, obesity and smoking, in a multivariable Mendelian randomization (MVMR) analysis.

**Method:**

Publicly available genome‐wide association study data were used for the genetic instrument of loneliness and five pleiotropic risk factors and AD. A two‐sample univariable MR model was tested for loneliness and AD risk followed by a multivariable MR model to include the additional five pleiotropic risk factors.

**Result:**

Univariable MR analysis indicated that genetic proxied risk of loneliness was not significantly associated with increased risk of AD (OR = 1.05, 95%CI: 0.72;3.65). In the multivariable model the estimate for loneliness (OR = 1.04, 95%CI:0.76;1.32) remained non‐significant. The pleiotropic paths of alcohol dependence (OR = 0.83, 95%CI:0.67;1.02), smoking (OR = 0.35, 95%CI:0.08;1.62), neuroticism (OR = 0.87, 95%CI:0.55;1.38), and education (OR = 0.56, 95%CI:0.21;1.54) were not significantly associated with increased risk for AD. However, higher body mass index (BMI) (OR = 0.41, 95%CI:0.19;0.87) appeared to reduce AD risk.

**Conclusion:**

The null findings reported here should be interpreted within the context of limitations inherent to MR models including, weak instrument bias and construct validity. Specifically, the loneliness measure may lack validity. Survivor bias may account for the unexpected direction of results for BMI. While our results suggest that most of the tested pathways were not significant, further research aimed at addressing the limitations of MR is needed to better understand the nuanced interplay between loneliness, pleiotropic risk factors, and AD risk.

